# Preparation of a novel zwitterionic striped surface thin-film composite nanofiltration membrane with excellent salt separation performance and antifouling property†

**DOI:** 10.1039/d0ra00480d

**Published:** 2020-04-23

**Authors:** Bo Lin, Huifen Tan, Wenchao Liu, Congjie Gao, Qiaoming Pan

**Affiliations:** Second Institute of Oceanography of the State Oceanic Administration Hangzhou 310012 China gaocj@zjut.edu.cn; Blue Star (Hangzhou) Membrane Industry Co., Ltd. Hangzhou 311106 China; Zhejiang University of Technology Hangzhou 310014 China

## Abstract

Thin-film composite (TFC) nanofiltration (NF) membranes were fabricated *via* the co-deposition of taurine, tannic acid (TA), and polyethyleneimine (PEI), followed by subsequent interfacial polymerization with trimesoyl chloride (TMC) on the surface of the polysulfone ultrafiltration substrates. The surface properties, including the roughness, hydrophilicity, surface potential, and NF performances were facilely tuned by varying the taurine content for the prepared TFC membranes. In addition, the as-prepared TFC NF membranes had an excellent antifouling property and flux recovery ratio (FRR) in humic acid (HA), bovine serum albumin (BSA) and sodium alginate (SA) filtration tests. These results also revealed that the taurine content controlled the formation of the striped surface. Thus, this work provided a viable strategy for fabricating TFC NF membranes with high selectivity and outstanding antifouling ability.

## Abstract 2

Thin-film composite (TFC) nanofiltration (NF) membranes with zwitterionic striped surface were fabricated *via* the co-deposition and interfacial polymerization.

## Graphical abstract

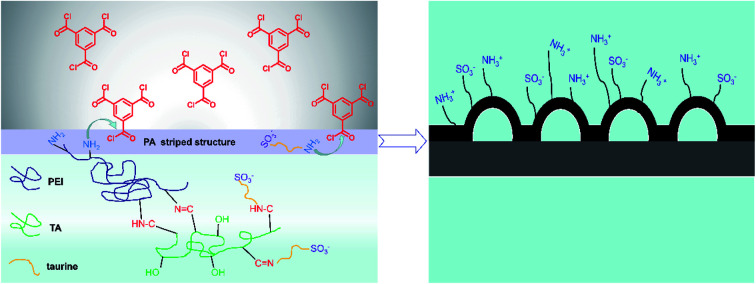

## 1 Introduction

Nanofiltration (NF) is a pressure-driven membrane separation technology with a molecular weight cut-off (MWCO) ranging from 200 to 1000 Da and a pore size of around 0.5–2.0 nm, which was confirmed as an effective technique for desalination and could also be used in liquid purification, sewage, and industrial wastewater treatments because of its low-pressure requirement.^1–4^ However, membrane fouling is now the bottleneck problem in membrane applications since the practical application conditions and separation systems of the membranes are far more complicated.^5^ For wastewater treatment, the membrane foulants mainly arise from macromolecular organic matter, humic substances, hydrocarbons, bacteria, suspended sludge and inorganic colloidal or mineral scaling, which inevitably lead to the unfavorable change of membrane structures and significant deterioration of the separation performance.^6,7^ Therefore, the construction of antifouling nanofiltration has a big significance in membrane applications.

In an antifouling membrane, advanced materials (such as silica spheres,^8^ nanoparticles,^9–12^ hydrophilic polymers,^13–15^ metallics^16,17^ as well as some functional carbon materials^18,19^) play an important role. Recently, polyphenol tannic acid (TA) has been widely used in membrane fabrication because of its abundance in plant tissue^20^ and versatility for membrane synthesis. Tannic acid has anti-bacterial, anti-enzymatic and astringent properties.^21^ With these properties, it is an ideal molecule for improving the antifouling performance. Kim *et al.*^22^ coated TA–Fe^III^ coordination complexes onto PES UF membrane surfaces *via* a facile one-pot assembly process to obtain desirable features, such as antifouling properties against proteins, oils, and microorganisms, as well as antimicrobial and heavy metal ion removal properties. Zhang *et al.*^23^ developed a facile TA-assisted *in situ* assembly approach during non-solvent induced phase separation (NIPS) to prepare antifouling NF membranes. Xu *et al.*^24^ reported on the addition of TA to a PVDF membrane for oil/water emulsion filtration to avoid membrane over-fouling. Aside from tannic acid, zwitterions have attracted much attention in the study of antifouling membranes owing to their high intrinsic hydrophilicity, which forms a hydration layer through the electrostatic interaction between the zwitterions and water molecules.^25^ This hydration layer provides a repulsive steric barrier to prevent the adsorption of organic molecules and bacteria on the substrate surface.^25^ Wang *et al.*^26^ introduced a zwitterionic copolymer carrying a positive charge into a negatively charged membrane *via* simple electrostatic adsorption in a one-step dip-coating process. During filtration with lysozyme for 1 h, the flux of this modified membrane was 93% of the initial flux, which was much higher than that of the original membrane. Davari *et al.*^27^ tethered the zwitterionic polyelectrolyte poly[1-vinyl-3(2-carboxyethyl)imidazolium betaine] (PVCIB) onto a commercial thin film composite polyamide (TFC PA) membrane, which exhibited remarkable antifouling ability to resist non-specific protein adsorption at neutral and alkaline pH values. However, both PA-PVI and PA-PVCIB membranes exhibited high resistance to the positively charged lysozyme adhesion under acidic pH conditions. In addition, the surface zwitterionization can be constructed on the membrane surface rather than adding a zwitterionic copolymer. Researchers used 1,3-propanesultone to create a zwitterionic surface by ring-opening directly on the nanofiltration membrane prepared from the interfacial polymerization of 3,3′-diamino-*N*-methyldipropylamine and 1,3,5-benzenetricarboxylic chloride (TMC).^28^ The zwitterionic *N*,*N*-bis(3-aminopropyl)methylamine was synthesized by Mi *et al.* to prepare an antifouling NF membrane with a 94.9% flux recovery ratio in BSA and SA solutions.^29^ The zwitterionic structures improved the hydrophilicity of the nanofiltration membrane, thus increasing the water permeability and antifouling performance to proteins and polysaccharides. Zwitterions and tannic acid have been widely applied in the preparation of antifouling membranes, while the effect of their combination still remains unknown.

Striped structures in the nanofiltration membrane were first illustrated by Tan *et al.* in 2018.^30^ The nanoscale spotted and striped Turing structures were generated by controlling the concentration of the PVA addition, which changes the diffusion rate of piperazine from the aqueous phase to the organic phase by hydrogen bond.^30,31^

In our study, taurine (3-amino-1-propanesulfonic acid) and polyethyleneimine were poured on the polysulfone (PSF) substrate. Taurine was introduced into the membrane to improve the antifouling performance, and tannic acid acted as a cross-linking agent to lock the zwitterion onto the membrane surface with catechol groups.^32^ Then, this chemical-stable anti-biofouling compound and polyethyleneimine (PEI) were used to build a hydrophilic zwitterionic striped structure membrane by interfacial polymerization with TMC. We first used the strategy in which we attached the hydrophilic material by chemical bond to build the striped structure, which obviously improved the membrane hydrophilicity. The influence of the molecule weight of PEI, the concentration of taurine, as well as the interfacial polymerization conditions on the membrane morphology, separation performance and anti-fouling property was explored. The as-prepared membranes were characterized by FTIR, SEM, EDX, XPS, AFM, contact angle goniometer and zeta potential measurement.

## 2 Experimental

### 2.1 Materials and reagents

The commercial PSF UF membrane (MWCO = 20 000 Da) used as the support membrane was provided by Hangzhou Water Treatment Technology Development Center (China). Taurine (3-amino-1-propanesulfonic, 98%), tannic acid (TA, 98%), polyethyleneimine (PEI, MW = 600, 1000, 1800, 2500, 10 000, 99%), polyethyleneimine (PEI, MW = 70 000, 50%), bovine serum albumin (BSA) and sodium alginate (SA) were purchased from Shanghai Macklin Biochemical Co., Ltd. The inorganic salts (*i.e.*, sodium phosphate tribasic dodecahydrate (Na_3_PO_4_·12H_2_O), sodium chloride (NaCl), magnesium chloride hexahydrate (MgCl_2_·6H_2_O), anhydrous sodium sulfate (Na_2_SO_4_) and anhydrous magnesium sulfate (MgSO_4_)) were provided from Hangzhou Lanbo Industrial Co., Ltd. Sodium dodecyl sulfate (SDS) and humic acid (HA) were purchased from Aladdin Industrial Corporation. Trimesoyl chloride (TMC, ≥98%) was purchased from Qingdao Benzo Chemical Co., China. Isopar L as an organic solvent was purchased from Exxon Mobil Corporation. Deionized (DI) water obtained by the electrodialysis of reverse osmosis water was used for all experiments. All chemical reagents were of analytical grade and implemented without further purification.

### 2.2 Fabrication of TFC NF membranes

TFC NF membranes were fabricated through a rapid co-deposition of PEI/TA/taurine onto the top surface of a PSF ultrafiltration (UF) substrate, followed by interfacial polymerization (IP) with TMC (Fig. S1†). The detailed fabrication process is illustrated in the ESI (S1).† The synthesized membranes are denoted as “M_*a*_”, where “*a*” is the weight concentration of taurine (example: 0.1% wt is written as 1). M_b_ was the membrane without crosslinking by TMC (Table 1).

**Table 1:** IP parameters corresponding to the labeled TFC NF membrane^a^

TFC membrane	TA content (wt%)	PEI content (wt%)	TMC content (w/v)	Taurine content (wt%)
M_0_	0.20	0.5	0.1	0
M_1_	0.20	0.5	0.1	0.1
M_2_	0.20	0.5	0.1	0.2
M_3_	0.20	0.5	0.1	0.3
M_4_	0.20	0.5	0.1	0.4
M_b_	0.20	0.5	0	0.2

a TA, PEI, taurine contents (wt%) are relative to the total IP solution volume (100 mL).

### 2.3 Characterization of the TFC NF membranes

All samples were dried in a vacuum oven at 40 °C for 12 h before characterization.

The functional structure of the TFC membrane surface was examined by attenuated total reflection Fourier transform infrared spectroscopy (ATR-FTIR, Nicolet 6700). For ATR-FTIR analysis of the membrane samples, the resulting NF membranes were characterized by the total reflection of a zinc selenide crystal. The chemical compositions of the NF membranes were measured by X-ray photoelectron spectroscopy (XPS, Kratos AXIS Ultra DLD). The morphologies of the NF membranes surface were observed by scanning electron microscopy (SEM, Hitachi S-4700). Then, the surface of the membranes was scanned after coating with gold. The NF membrane surface roughness was obtained by atomic force microscopy (AFM, XE-100) with 5 μm × 5 μm scanning range. RMS means the root mean square roughness, and *R*_a_ means the arithmetic mean roughness. The water contact angle was measured on a dried membrane surface by a contact angle analyzer (OCA-20, Germany) at ambient temperature. After each drop, the image was captured and the reading was recorded. For each sample, at least 12 readings were obtained, and the average contact angle values were calculated from these readings. Zeta potentials of the membrane samples were measured by a SurPASS analyzer (Anton Paar, Austria). The test reagent used for our study was 0.001 mol L^−1^ KCl, and the pH value was varied to investigate its effect on the zeta potential.^33^

### 2.4 Membrane performance evaluation

The membrane performance was tested using a laboratory-scale cross-flow apparatus with an effective area of 12.6 cm^2^ at 0.5 MPa and 25 ± 1 °C. Before the test, each membrane was pressurized with DI water at 0.75 MPa and 25 ± 1 °C for 30 min to reach a steady state. Various salts (Na_2_SO_4_, NaCl, MgSO_4,_ and MgCl_2_) in aqueous solutions with a concentration of 1 g L^−1^ were used as the feed solution to evaluate the separation performance of the membrane. The flux (*J*, L m^−2^ h^−1^) and rejection (*R*, %) were calculated according to eqn (1) and (2), respectively:1
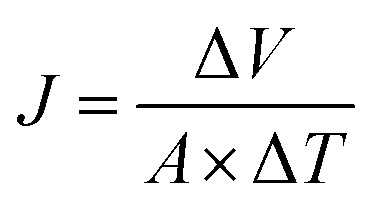
where Δ*V* (L), *A* (m^2^) and Δ*T* (h) correspondingly represent the volume of the permeate, the effective membrane area and the time for permeate collection.2
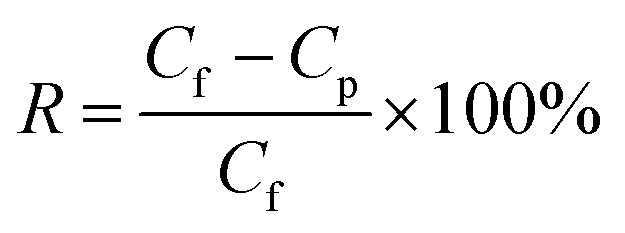
where *C*_p_ (mol L^−1^) and *C*_f_ (mol L^−1^) refer to the concentration of permeate and feed solution, respectively.

### 2.5 Antifouling property of the membrane

The antifouling property of the membrane was investigated by using the organic foulant (BSA, SA, and HA) solution (500 mg L^−1^) in the same cross-flow apparatus at 25 ± 1 °C.^34^ The membranes were compacted with DI water at 0.5 MPa and 25 ± 1 °C for 30 min to achieve a stable state, and the pure water flux of the original membrane (*J*_0_) was measured for 3 h at 0.5 MPa. Then, the fouling experiment was carried out with 100 mg L^−1^ BSA, SA or HA aqueous solution as the feed solution for another 10 h, and the steady value of the flux was noted as *J*_1_. The normalized flux *J*_1_/*J*_0_ refers to the flux declining ratio during the fouling process. After that, the fouled membrane was thoroughly flushed by DI water for 30 min at 0.05 MPa. Finally, the pure water flux (*J*_2_) of the cleaned membrane was measured for 3 h at 0.5 MPa. The above procedure was repeated 2 times to evaluate the flux recovery ratio (FRR), which was determined by eqn (3).3
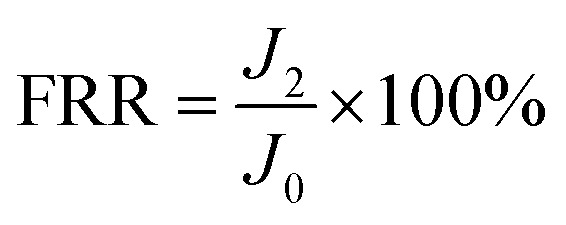


The total fouling ratio (*R*_t_), reversible fouling ratio (*R*_r_) and irreversible fouling ratio (*R*_ir_) were calculated by eqn (4)–(6), respectively.4
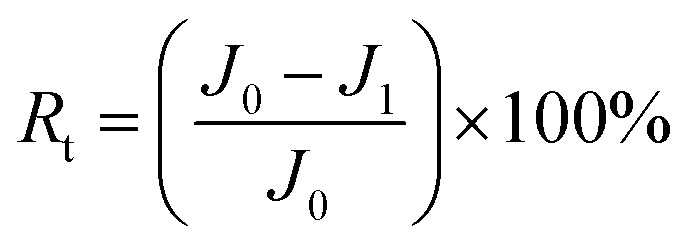
5
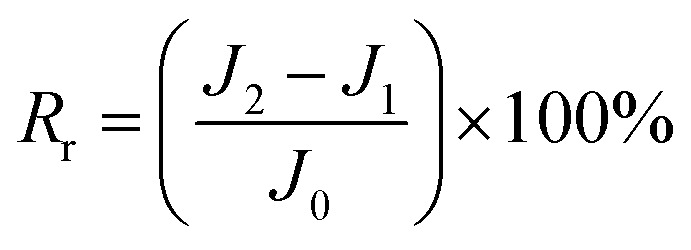
6
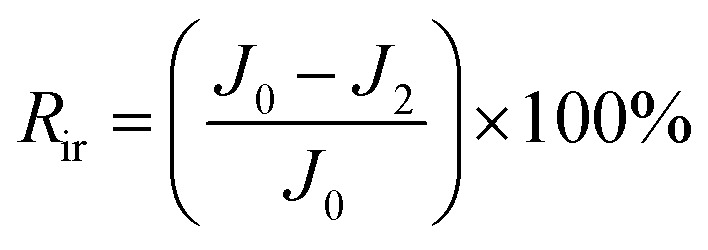


## 3 Results and discussion

### 3.1 Characterization of TFC NF membranes

#### 3.1.1 Physical and chemical structures of the TFC membranes

As shown in Fig. 2, the chemical components of the membrane surfaces were characterized by ATR-FTIR. The specific absorption peak at 1039 cm^−1^ was denoted as the SO_3_^−^ groups that were observed in M_2_ because of the introduction of taurine.^35^ The specific absorption peaks at 1658 cm^−1^ and 1530 cm^−1^ were attributed to the existence of the formation of –C=O (amide I and amide II).^36,37^ With the increase in the concentration of taurine, the intensity of this peak was decreased. The band appearing in the M_b_ and M_2_ membrane spectra at 1645 cm^−1^ was assigned to the formation of the –C=N bond.^38,39^ The region from 2800 cm^−1^ to 2900 cm^−1^ in the blue circle was assigned to the specific absorption peak of the primary and secondary amines.^40^ The absorption bands of –OH and N–H were assigned at around 3429 cm^−1^ in the green circle.^41^ Due to the crosslinking by TMC, the broad peak became weaker in M_0_ and M_2_. M_0_ and M_2_ showed a newly emerged broad peak at around 1725 cm^−1^ owing to the stretching vibrations of the ester groups (–COO) provided by the crosslinking of TA and TMC.^42^ Furthermore, the XPS spectrum is shown in Fig. 3. When taurine was introduced, the intensity of the S 2s and S 2p peaks increased, which confirmed that the taurine was locked into the membrane surface. In addition, the XPS C 1s and N 1s spectra in Fig. 4 were further used to prove that the –C=N bond was formed during the membrane immersion in the aqueous phase,^39^ and the C–S bond was confirmed to be introduced by comparing the species of M_0_ and M_2_. Table 2 displays the elemental information of various TFC membranes, which was calculated from the corresponding X-ray photoelectron spectroscopy (XPS) spectra in Fig. 3. The ratio of C/O and C/N increased from 3.50 to 3.74 as the amounts of taurine varied from 0 to 0.3%, which was caused by the introduction of the C and N atoms from taurine. The C/O and C/N declined when the taurine content reached 0.4%, which was caused by the reaction of free taurine and TMC. The remaining taurine that did not attach to TA reacted with TMC directly to decrease the surface degree of crosslinking. The change of the C/O and C/N ratio in M_4_ gave evidence for the reaction of taurine and TMC. Therefore, the results from ATR-FTIR and XPS demonstrated that the prepared membrane had a zwitterionic surface and the reaction process in IP.

**Fig. 1:**
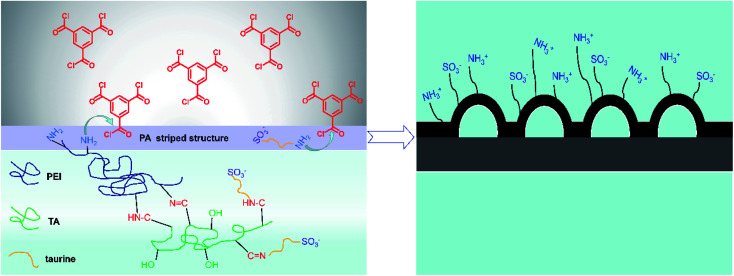
The mechanism of interface polymerization and zwitterionic striped structure formation.

**Fig. 2:**
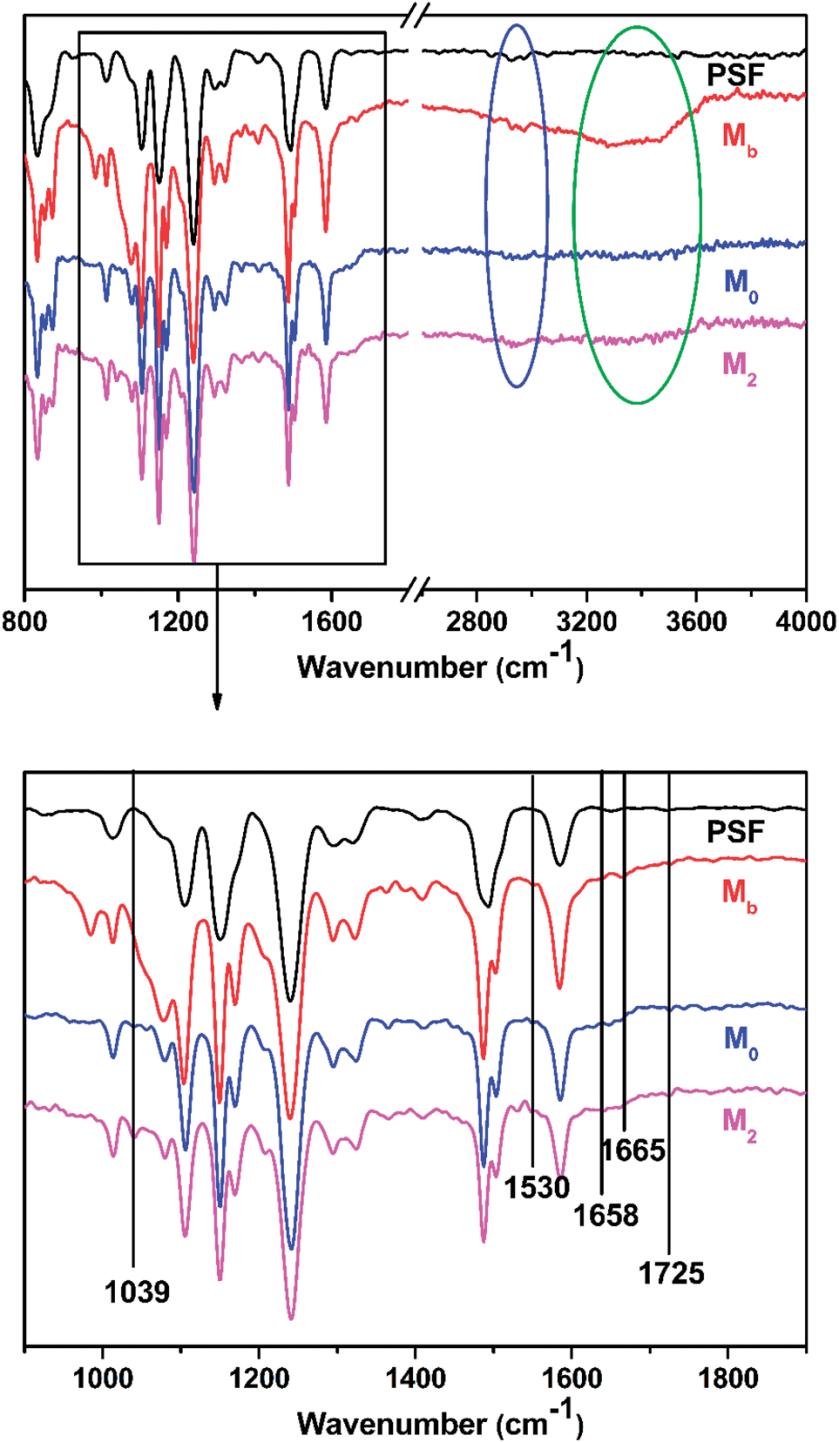
The ATR-FTIR spectra of the PSF support membrane, M_0_, M_2_, and M_b_.

**Fig. 3:**
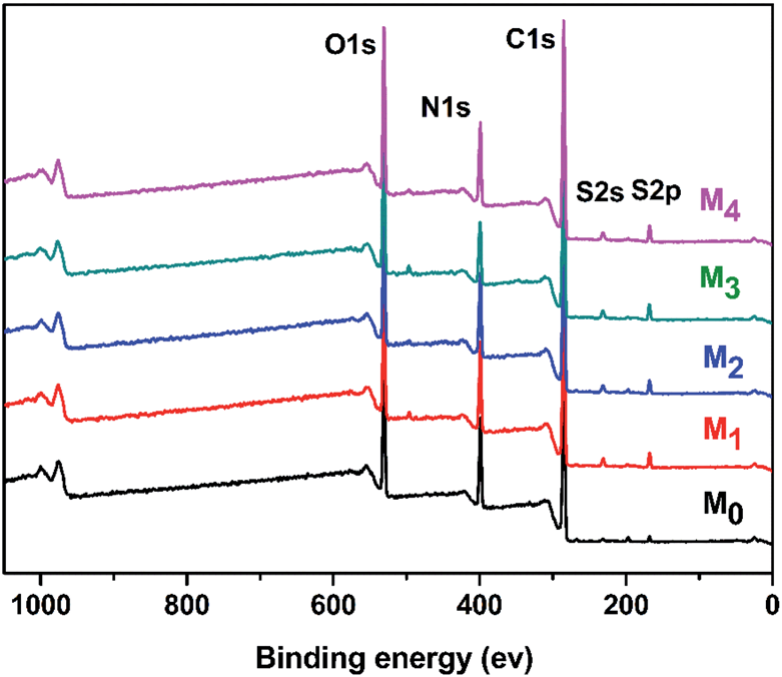
Wide-scan XPS spectrum of the TFC membranes.

**Fig. 4:**
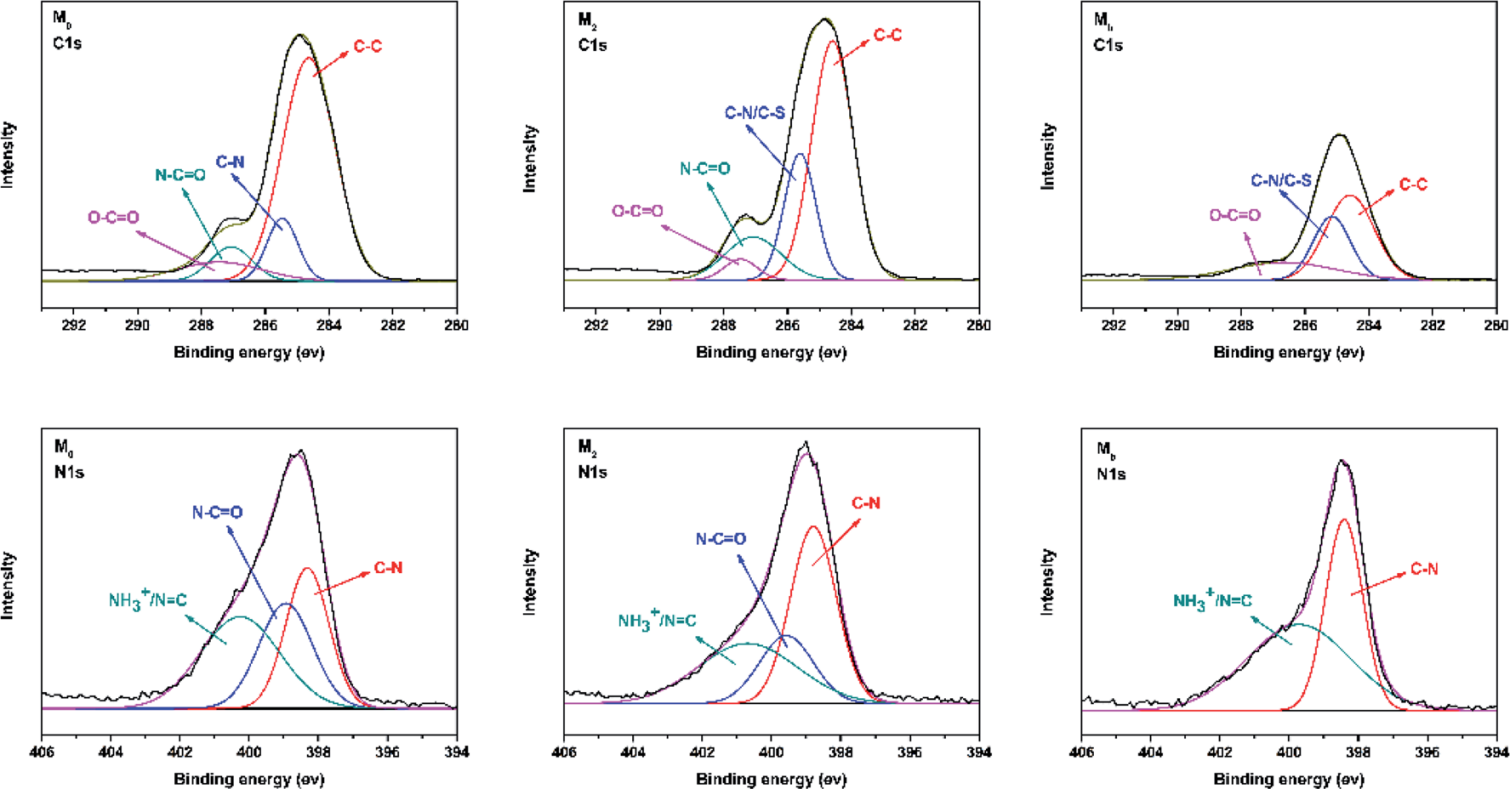
Deconvoluted C 1s and N 1s XPS spectra for membranes including M_0_, M_2_, and M_b_.

**Table 2:** Chemical compositions of various TFC membranes

Membrane	C (%)	N (%)	O (%)	S (%)	Cl (%)	C/O	C/N
M_0_	63.84	17.07	18.26	0.81	0.02	3.50	3.74
M_1_	64.09	15.77	18.17	1.96	0.01	3.53	4.06
M_2_	65.05	14.85	17.98	2.11	0.01	3.62	4.38
M_3_	66.60	13.22	17.80	2.36	0.02	3.74	5.03
M_4_	63.49	15.77	18.23	2.50	0.01	3.48	4.03
M_b_	61.01	18.78	18.43	1.78	0	3.31	3.25

#### 3.1.2 Surface and cross-section morphologies of the membranes

**Fig. 5:**
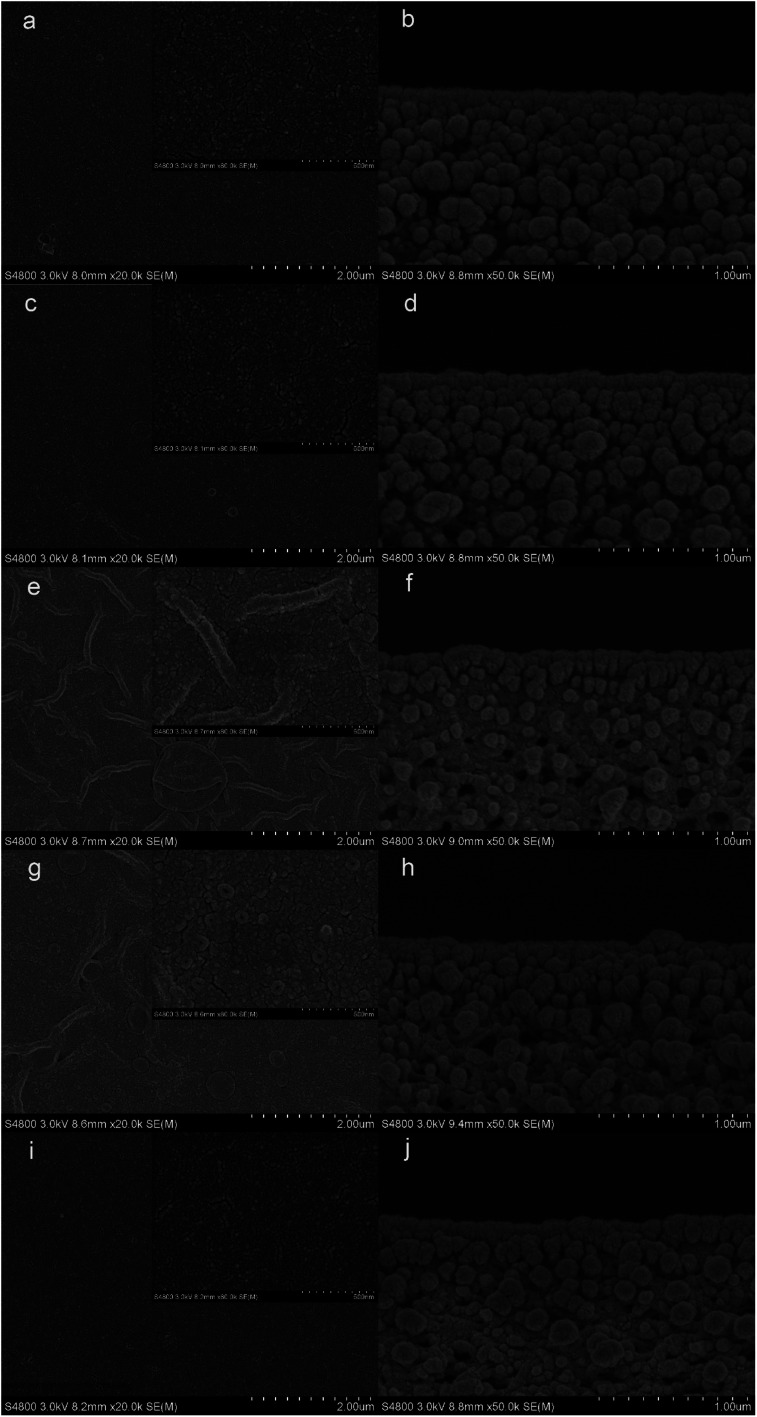
SEM images of the TFC membranes: M_0_ ((a) surface, (b) cross-section), M_1_ ((c) surface, (d) cross-section), M_2_ ((e) surface, (f) cross-section), M_3_ ((g) surface, (h) cross-section), M_4_ ((i) surface, (j) cross-section).

The top surface and cross-section morphologies of each membrane were observed under SEM. From Fig. 5, the nanostrand hybrid morphology appeared in panels (e) and (g). The surface morphology was changed by the contents of the additional taurine. When 0.2% or 0.3% taurine was added, the roughness and surface area of the striped structure increased (as shown in Table 3).^30^ The membrane surface property and hydrophilicity, in particular, were improved with increasing taurine content. Also, the reaction of PEI, TA, and taurine helped the hydrophilic groups attach to PEI, which hindered the further diffusion of the PEI monomer and efficiently reduced the polyamide thickness.^43^ These hydrophilic groups also absorbed water to form a water layer to change the surface tension between the aqueous phase and organic phase in the IP reaction to create the striped structure^44^ (Fig. 1). Fig. 1 illustrates that the sulfonic acid group was much more hydrophilic, which caused the PEI to remain at the aqueous phase for as long as possible, and increased the surface tension. So, the striped structure appeared. It is important to highlight that the striped structure disappeared when a larger amount of the taurine was added (0.4%). The IP reaction takes place in the organic phase,^45^ and the micromolecules were easier to diffuse to the organic phase from the aqueous phase than the macromolecules.^30^ Thus, the redundant taurine molecules (not combined with TA) were easily diffused to the organic phase to immediately react with TMC, and the reaction between PEI and TMC was further decreased. Moreover, the cross-section structure showed that the degree of crosslinking was decreased by the increased taurine content.^46^ Thus, a loose surface morphology could enhance the permeability of the water molecule.^47^ The roughness of the membranes is depicted by AFM in Table 3. The *R*_a_ and RMS of M_2_ were 4.61 nm and 5.97 nm, respectively. After the taurine was introduced, much rougher surfaces with more nodular structures were observed for membranes M_1_ through M_4_ in comparison with M_0_. The *R*_a_ values of membranes M_1_ through M_4_ were 3.54 nm, 4.61 nm, 5.64 nm, and 7.17 nm, respectively. These results proved the conclusions from the previously discussed SEM images that the formation of the striped structures increased the surface roughness. Therefore, the surface morphology and roughness, which are related to the permeability and anti-fouling property of the membrane, were dominated by the introduction of taurine.

**Table 3:** AFM roughness parameters of the TFC membrane surfaces. Surface roughness values of the 5 × 5 μm^2^ sample scans are represented as *R*_a_ (average roughness), RMS (root mean square roughness), and *R*_m_ (maximum height)

TFC membrane	*R*_a_ (nm)	RMS (nm)	*R*_m_ (nm)
M_0_	3.39	4.29	43.7
M_1_	3.54	4.54	48.8
M_2_	4.61	5.97	63.5
M_3_	5.64	7.53	80.6
M_4_	7.17	9.59	108

#### 3.1.3 Water contact angle and surface charge of the membranes

**Fig. 6:**
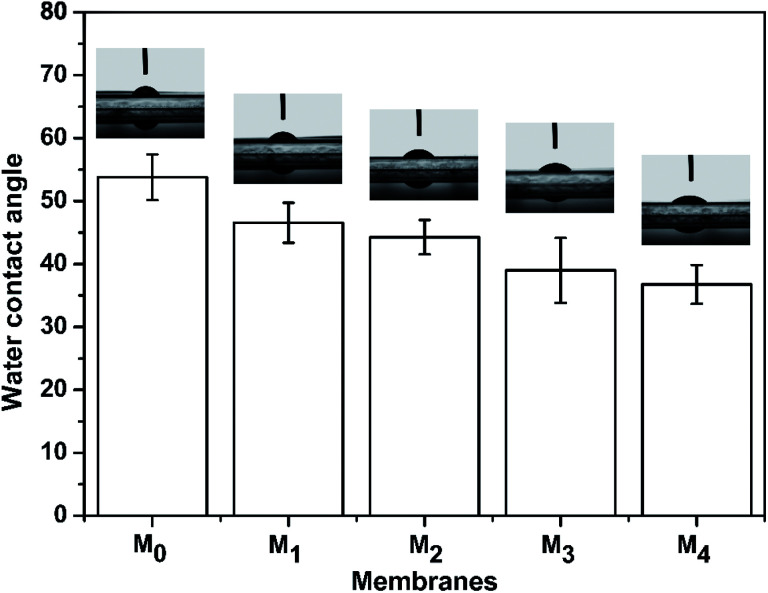
The water contact angle of the M_0_, M_1_, M_2_, M_3_, and M_4_ membranes.

**Fig. 7:**
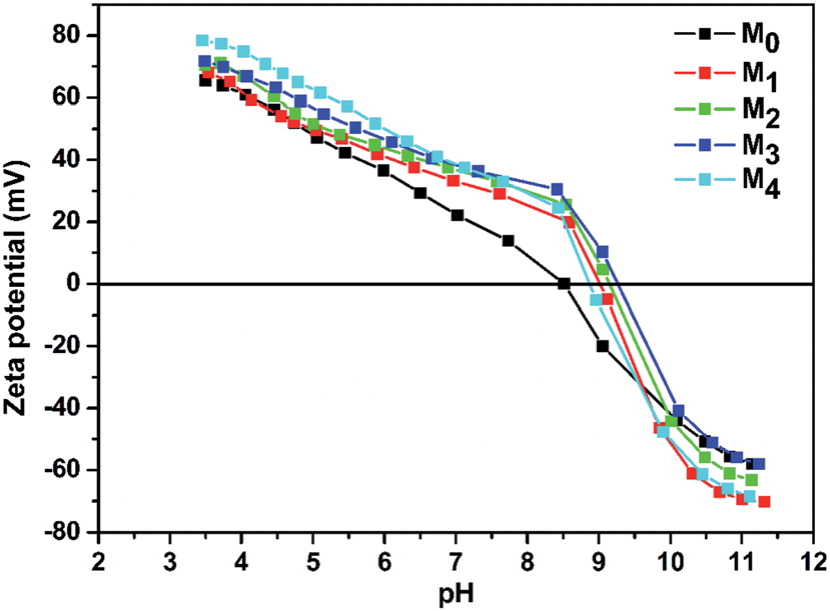
Membrane surface charges as a function of pH.

The water contact angles of the TFC membranes are shown in Fig. 6. It is intuitive that the water contact angle of the membrane was decreased by the increase of the taurine content. Amine groups and sulfonic acid groups have a strong affinity to water, which increased the surface hydrophilicity of the membrane.^48,49^ Thus, the change of the water contact angle demonstrated that the introduction of taurine improved the surface hydrophilicity, corresponding to the result in Table 3. In addition, the surface charge of the membranes was evaluated by the zeta potential test. The result is displayed in Fig. 7. All membrane surfaces were found to be positively charged when the pH value was at 6.5 (test value) because the polymerization between PEI and TMC led to a polyamide thin-film with a large number of unreacted positive primary and secondary amine groups.^50^ When the concentration of taurine increased from 0 to 0.3%, the isoelectric point was shifted to a higher pH value. One reason is that the increased taurine replaced some amino groups of PEI to react with TA and TMC to release more amino groups of PEI. Meanwhile, taurine itself brought in amino groups. Nevertheless, when the taurine content came to 0.4%, the isoelectric point was shifted to the left. The explanation for this result was that the high concentration of the taurine strongly impeded the reaction between TA and PEI or TMC and PEI (Fig. 1). These unreacted PEIs were washed by the water, causing the amino groups in the membrane surface to decrease. Although the surface had become more positively charged with the introduction of taurine, the sulfonic acid groups still provided negative charges in the membrane to form a zwitterionic surface that could not be evaluated by the surface zeta potential test.^51^

### 3.2 Membrane performance

The fabrication conditions were evaluated during the preparation process, and the influence of these conditions on membrane performance are described below.

#### 3.2.1 Concentration of taurine

The effect of the taurine content on the resulting membrane separation performance are presented in Fig. S2(a).† The reaction time in the aqueous phase and TMC organic phase was 3 min and 60 s, respectively, and the PEI molecular weight was 70 000, as listed in Table 1. When the taurine content varied from 0 to 0.4%, the rejection of Na_2_SO_4_ and NaCl increased in the first stage, and subsequently decreased. Furthermore, when the taurine content was at 0.2%, the separation performance of Na_2_SO_4_ and NaCl was superior and kept a relatively high MgCl_2_ rejection. The membrane containing 0.2% taurine with a striped surface also had a larger water contact area,^30^ which had a considerable water flux. The NF membrane is considered as a charged porous film, and there are two main factors that affect the rejection of the NF membrane that coincides with the Donnan exclusion theory and steric hindrance effect: the surface charge plays a crucial role.^52^ With the addition of taurine, the surface property of the membrane was changed. The high Na_2_SO_4_ rejection and the reduction of MgCl_2_ rejection can be rationalized by the Donnan effect and size exclusion effect, as there was a charge shielding effect in the charged membrane surface and the hydration radii of Na^+^ and Cl^−^ are too small to be rejected.^53^ The sulfonic acid groups were taken into the membrane with the unreacted amino groups of PEI to construct a zwitterionic surface, which altered the surface potential. The taurine content increased, enhancing the negative potential, so the rejection of Na_2_SO_4_ first increased. On the other hand, the taurine content dominated the surface menology. When the taurine was introduced, the taurine that did not combine with TA easily reacted with TMC to decrease the degree of surface crosslinking to form a thinner PA layer. This is why the rejection of Na_2_SO_4_, MgCl_2_, and NaCl was reduced when the taurine content increased to more than 0.2%, and the flux of the membrane rose as a result of the increased taurine content. Thus, the membrane with 0.2% taurine exhibited relatively optimistic separation performance and water permeability.

#### 3.2.2 PEI molecular weight

The influence of the PEI molecular weight is presented in Fig. S2(b).† The reaction times in the aqueous phase and TMC organic phase were 3 min and 60 s, respectively, and the taurine content was 0.2%. When the molecular weight increased from 600 to 2500, the rejection of Na_2_SO_4_, MgCl_2_ and NaCl increased. When the molecular weight was further enlarged, the rejection of NaCl was decreased. The water flux also declined with the increased molecular weight of PEI. The reason may be that the selective layer became more dense with the increased molecular weight of PEI.^39^ In addition, a PEI molecule with a larger molecular weight has more amino groups to provide more positive charges for a high divalent cation rejection.^52^ In addition, the larger molecular weight (10 000 and 70 000) PEI was harder to introduce to the organic phase to react with TMC, but provided more amino groups, which explained the stable Na_2_SO_4_ rejection and rapid decline of the NaCl rejection.

#### 3.2.3 Co-deposition time

The role of the co-deposition time in membrane performance is displayed in Fig. S2(c).† The reaction time in the TMC organic phase was 60 s. The taurine content was 0.2% and the PEI molecular weight was 70 000. When the time varied from 1 min to 3 min, the water permeability was improved and the rejection of Na_2_SO_4_, MgCl_2_, and NaCl was increased. That may be ascribed to the deposition of more hydrophilic monomers, especially the amino groups of PEI, in the aqueous phase. After extending the deposition time from 3 min to 9 min, the water permeability of the membrane dropped from 57.58 L m^−2^ h^−1^ to 43.82 L m^−2^ h^−1^. The explanation for this phenomenon was that much more hydroxyl groups of TA reacted with the amino groups to form a crosslinking structure, which weakened the surface permeability of the membrane. The rejection of Na_2_SO_4_ and NaCl showed no great changes, while the rejection of MgCl_2_ was decreased since the rejection of salt in the TFC membranes was mainly dominated by the surface charge rather than the surface structure,^39^ and the amino groups do not have much impact on the divalent rejection.

#### 3.2.4 IP reaction time

The effect of the IP reaction time is illustrated in Fig. S2(d).† The reaction time in the aqueous phase was 60 s. The taurine content was 0.2% and the PEI molecular weight was 70 000. When the reaction time was 3 min, the Na_2_SO_4_ rejection was >97%, as the reaction rate of the interface polymerization was very fast.^54^ When the reaction time rose from 30 s to 60 s, the rejection of Na_2_SO_4_, MgCl_2_ and NaCl increased rapidly, while the water flux decreased rapidly. When the reaction time increased from 60 s to 150 s, the Na_2_SO_4_ rejection became steady while that of MgCl_2_ decreased, and the NaCl rejection still increased rapidly and the declination tendency of the water flux slowed down. That can be ascribed to the increase of the degree of crosslinking with the expanding reaction time. Therefore, the best condition for the membrane preparation was when the reaction times in the aqueous phase and TMC organic phase were 3 min and 60 s, respectively, the taurine content was 0.2% and the PEI molecular weight was 70 000, which were the preparation conditions of M_2_.

#### 3.2.5 The membrane performance of M_0_ and M_2_ in four different salt solutions

**Fig. 8:**
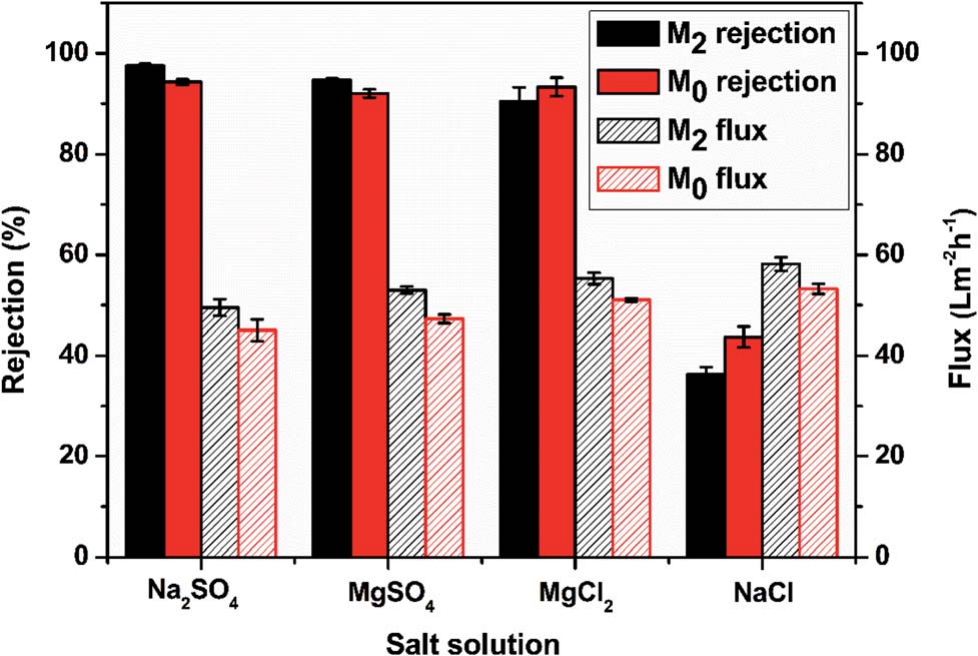
The membrane performance of M_0_ and M_2_ in four different salt solutions.

Fig. 8 displays the membrane performance of M_0_ and M_2_ in four different salt solutions at 1000 ppm. For M_2_, the rejections of Na_2_SO_4_, MgSO_4_, MgCl_2_ and NaCl were 97.55%, 94.72%, 90.40%, and 36.36%, respectively. For M_0_, the rejections of Na_2_SO_4_, MgSO_4_, MgCl_2,_ and NaCl were 94.29%, 92.00%, 93.32%, and 43.70%, respectively. The rejection mechanism of the nanofiltration membranes was mainly determined by the molecular size (sieve effect) and the charge effect.^55^ The M_2_ membrane with the zwitterionic surface had a special charge distribution for divalent ion rejection. The sulfonic acid group helped to increase the rejection of SO_4_^2−^ and maintain the relatively high rejection of Mg^2+^. In addition, the water flux of M_2_ in these four salt solutions was 49.53 L m^−2^ h^−1^, 52.97 L m^−2^ h^−1^, 55.3 L m^−2^ h^−1^, and 58.16 L m^−2^ h^−1^, respectively. The striped surface increased the surface water contact area to result in a relatively higher water flux. This result showed that M_2_ had a better salt separation performance and water permeability than M_0_.

### 3.3 Antifouling performance of the TFC membrane

Generally, nanofiltration membranes easily suffer from membrane fouling in applications, which restricts the lifetime of the membrane. Factors like the electrostatic interaction, hydrogen bonding effect, hydrophobic impact, and van der Waals forces normally have a great impact on membrane fouling.^56^

**Fig. 9:**
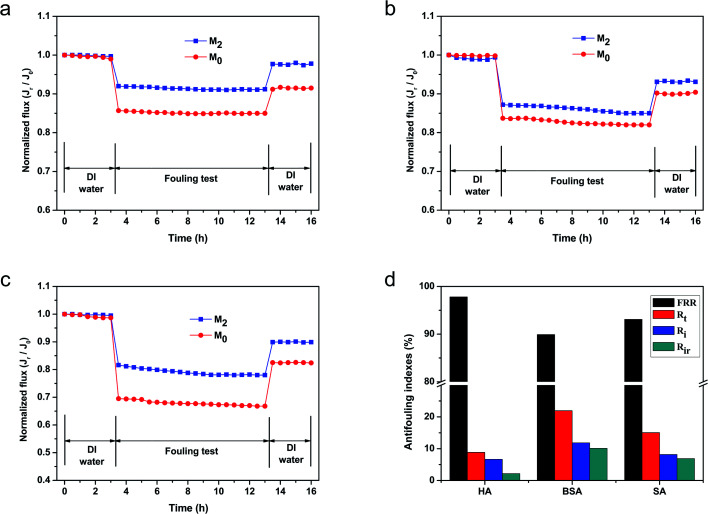
Time-dependent flux for the M_2_ and M_0_ membranes during the filtration of foulants at 500 ppm: (a) HA, (b) SA, (c) BSA, and the (d) antifouling indices of M_2_ in these foulants.

A comparison of the antifouling performances of M_2_ and M_0_ is shown in Fig. 9. Fig. 9(a)–(c) display the antifouling performances of M_2_ and M_0_ at pH = 7 in HA, SA and BSA solutions of 500 ppm, respectively. When the feed solution was replaced by a model foulant solution, the water flux of both membranes declined due to the extra osmotic resistance caused by the deposition of foulants on the membrane surface. Fig. 9(a) shows that the flux of M_0_ decreased rapidly and reached a quasi-steady stage of 85% to the initial water flux, while M_2_ showed a lower flux reduction ratio (9%) in the HA solution. After being cleaned with DI water, the relative water FRRs of M_0_ and M_2_ were 91.5% and 97.8%, respectively. These flux variations during the antifouling test indicate that the M_2_ membrane had an excellent humic acid antifouling performance. In addition, Fig. 9(b) shows the antifouling performances of M_0_ and M_2_ in a 500 ppm SA solution. The flux of M_0_ decreased rapidly and reached a quasi-steady stage of 82% to the initial water flux and the flux reduction ratio was 10% after being cleaned by DI water, while M_2_ showed a relatively lower flux reduction ratio (7%).

Moreover, the antifouling performances of M_2_ and M_0_ in the BSA solution are shown in Fig. 9(c). The permeation flux of M_2_ and M_0_ experienced a marked decrease in 78% and 67%, respectively, demonstrating severe BSA membrane fouling. After being cleaned with DI water, the water permeabilities of these membranes recovered to 90% and 82%, which were much lower than the original pure water flux. The lower recoveries indicated that these membranes had a relatively low flux recovery and reversible fouling. These results were primarily ascribed to the introduction of the sulfonic acid groups on the membrane surface. As we all known, the BSA, HA and SA foulants used in this experiment were all negatively charged under the neutral filtration condition (pH = 7).^12^ The introduction of the sulfonic acid groups provided more surface negative charges, which hindered the foulant molecules from contacting the membrane surfaces *via* electrostatic interactions. Meanwhile, the sulfonic acid groups increased the surface hydrophilicity and helped absorb water molecules. These changes enabled the formation of a hydration layer that prevented the adsorption of organic foulants on the membrane surface due to the higher maximum Gibbs free energy than the unmodified surface.^57,58^ Although the roughness of M_2_ was greater than M_0_ owing to it theoretically being embedded with more contaminants, the surface hydration layer was dense enough to cover the surface gap and help eliminate the influence of the membrane surface roughness. In addition, the zwitterionic surface provides some electrostatic resistance to these charged foulants. Thus, the M_2_ membrane showed better antifouling property than M_0_. Four parameters (FRR, *R*_t_, *R*_r_, and *R*_ir_) were utilized to evaluate the antifouling performance of M_2_ as summarized in Fig. 9(d). The FRR in the HA, BSA, and SA filtration test was 97.8%, 89.9%, and 93.1%, respectively. These results demonstrated that M_2_ showed remarkable antifouling properties in the HA solution, while having relatively lower FRR in the BSA and SA solutions. M_2_ also had a relatively better antifouling property in the HA solution. The explanation for this was that the BSA and SA solutions had relatively higher molecular weights and stronger viscidity values than HA. Therefore, the prepared membrane M_2_ demonstrated excellent performance in the membrane fouling evaluation.

## 4 Conclusion

In summary, novel TFC NF membranes with zwitterionic striped surfaces were fabricated *via* the co-deposition of taurine, TA, and PEI onto the PSF ultrafiltration membrane, followed by IP with TMC. The skin layer of the membranes was dense and defect-free; this endowed the TFC membranes with a high rejection (over 90%) for multivalent ions. The pure water flux of the membrane was also more than 61.28 L m^−2^ h^−1^ because of the striped structure. We first used hydrophilic micromolecules to change the diffusion rate of the macromolecules by chemical bonding rather hydrogen bond. In addition, the membrane showed a favorable antifouling property in HA, SA and BSA solutions (FRR > 89%). In conclusion, we propose a novel and effective strategy for promoting both the structural and chemical properties of TFC NF membranes to meet the antifouling demands of practical filtration processes, giving insight into the construction of a zwitterionic striped surface membrane by the introduction of micromolecules to macromolecules in the aqueous phase *via* chemical bonds.

## Conflicts of interest

The authors declared that they have no conflicts of interest to this work. We declare that we do not have any commercial or associative interest that represents a conflict of interest in connection with the work submitted.

## Supplementary Material

RA-010-D0RA00480D-s001

## References

[cit1] Székely G., Bandarra J., Heggie W., Sellergren B., Ferreira F. C. (2011). Organic solvent nanofiltration: a platform for removal of genotoxins from active pharmaceutical ingredients. J. Membr. Sci..

[cit2] Cheng J., Shi W., Zhang L., Zhang R. (2017). A novel polyester composite nanofiltration membrane formed by interfacial polymerization of pentaerythritol (PE) and trimesoyl chloride (TMC). Appl. Surf. Sci..

[cit3] Cheng X. Q., Shao L., Lau C. H. (2015). High flux polyethylene glycol based nanofiltration membranes for water environmental remediation. J. Membr. Sci..

[cit4] Koros W. J., Zhang C. (2017). Materials for next-generation molecularly selective synthetic membranes. Nat. Mater..

[cit5] Rana D., Matsuura T. (2010). Surface Modifications for Antifouling Membranes. Chem. Rev..

[cit6] Zhao X., Zhang R., Liu Y., He M., Su Y., Gao C., Jiang Z. (2018). Antifouling membrane surface construction: chemistry plays a critical role. J. Membr. Sci..

[cit7] Li X., Liu C., Yin W., Chong T. H., Wang R. (2019). Design and development of layer-by-layer based low-pressure antifouling nanofiltration membrane used for water reclamation. J. Membr. Sci..

[cit8] Ang M. B. M. Y., Pereira J. M., Trilles C. A., Aquino R. R., Huang S.-H., Lee K.-R., Lai J.-Y. (2019). Performance and antifouling behavior of thin-film nanocomposite nanofiltration membranes with embedded silica spheres. Sep. Purif. Technol..

[cit9] Ang M. B. M. Y., Ji Y.-L., Huang S.-H., Lee K.-R., Lai J.-Y. (2019). A facile and versatile strategy for fabricating thin-film nanocomposite membranes with polydopamine-piperazine nanoparticles generated in situ. J. Membr. Sci..

[cit10] Bagheripour E., Moghadassi A. R., Hosseini S. M., Ray M. B., Parvizian F., Van der Bruggen B. (2018). Highly hydrophilic and antifouling nanofiltration membrane incorporated with water-dispersible composite activated carbon/chitosan nanoparticles. Chem. Eng. Res. Des..

[cit11] Abdelsamad A. M. A., Khalil A. S. G., Ulbricht M. (2018). Influence of controlled functionalization of mesoporous silica nanoparticles as tailored fillers for thin-film nanocomposite membranes on desalination performance. J. Membr. Sci..

[cit12] You X., Ma T., Su Y., Wu H., Wu M., Cai H., Sun G., Jiang Z. (2017). Enhancing the permeation flux and antifouling performance of polyamide nanofiltration membrane by incorporation of PEG-POSS nanoparticles. J. Membr. Sci..

[cit13] An Q., Li F., Ji Y., Chen H. (2011). Influence of polyvinyl alcohol on the surface morphology, separation and anti-fouling performance of the composite polyamide nanofiltration membranes. J. Membr. Sci..

[cit14] Cheng J., Zhang Z., Shi W., Zhang R., Zhang B., Bao X., Guo Y., Cui F. (2018). A novel polyester composite nanofiltration membrane prepared by interfacial polymerization catalysed by 4-dimethylaminopyridine: enhanced the water permeability and anti-fouling ability. Polymer.

[cit15] Wu J., Wang Z., Wang Y., Yan W., Wang J., Wang S. (2015). Polyvinylamine-grafted polyamide reverse osmosis membrane with improved antifouling property. J. Membr. Sci..

[cit16] Li R., Wu Y., Shen L., Chen J., Lin H. (2018). A novel strategy to develop antifouling and antibacterial conductive Cu/polydopamine/polyvinylidene fluoride membranes for water treatment. J. Colloid Interface Sci..

[cit17] Abdi G., Alizadeh A., Zinadini S., Moradi G. (2018). Removal of dye and heavy metal ion using a novel synthetic polyethersulfone nanofiltration membrane modified by magnetic graphene oxide/metformin hybrid. J. Membr. Sci..

[cit18] Anand A., Unnikrishnan B., Mao J.-Y., Lin H.-J., Huang C.-C. (2018). Graphene-based nanofiltration membranes for improving salt rejection, water flux and antifouling – a review. Desalination.

[cit19] Thebo K. H., Qian X., Zhang Q., Chen L., Cheng H. M., Ren W. (2018). Highly stable graphene-oxide-based membranes with superior permeability. Nat. Commun..

[cit20] Picchio M. L., Linck Y. G., Monti G. A., Gugliotta L. M., Minari R. J., Alvarez Igarzabal C. I. (2018). Casein films crosslinked by tannic acid for food packaging applications. Food Hydrocolloids.

[cit21] Zhang Y., Su Y., Peng J., Zhao X., Liu J., Zhao J., Jiang Z. (2013). Composite nanofiltration membranes prepared by interfacial polymerization with natural material tannic acid and trimesoyl chloride. J. Membr. Sci..

[cit22] Kim H. J., Kim D.-G., Yoon H., Choi Y.-S., Yoon J., Lee J.-C. (2015). Polyphenol/Fe^III^ Complex Coated Membranes Having Multifunctional Properties Prepared by a One-Step Fast Assembly. Adv. Mater. Interfaces.

[cit23] Zhang R., He M., Gao D., Liu Y., Wu M., Jiao Z., Su Y., Jiang Z. (2018). Polyphenol-assisted in situ assembly for antifouling thin-film composite nanofiltration membranes. J. Membr. Sci..

[cit24] Xu L., He Y., Feng X., Dai F., Yang N., Zhao Y., Chen L. (2018). A comprehensive description of the threshold flux during oil/water emulsion filtration to identify sustainable flux regimes for tannic acid (TA) dip-coated poly(vinylidene fluoride) (PVDF) membranes. J. Membr. Sci..

[cit25] Yang Y., Ramos T. L., Heo J., Green M. D. (2018). Zwitterionic poly(arylene ether sulfone) copolymer/poly(arylene ether sulfone) blends for fouling-resistant desalination membranes. J. Membr. Sci..

[cit26] Wang S.-Y., Fang L.-F., Cheng L., Jeon S., Kato N., Matsuyama H. (2018). Improved antifouling properties of membranes by simple introduction of zwitterionic copolymers via electrostatic adsorption. J. Membr. Sci..

[cit27] Davari S., Omidkhah M., Abdollahi M. (2018). Improved antifouling ability of thin film composite polyamide membrane modified by a pH-sensitive imidazole-based zwitterionic polyelectrolyte. J. Membr. Sci..

[cit28] Mi Y.-F., Zhao F.-Y., Guo Y.-S., Weng X.-D., Ye C.-C., An Q.-F. (2017). Constructing zwitterionic surface of nanofiltration membrane for high flux and antifouling performance. J. Membr. Sci..

[cit29] Mi Y.-F., Xu G., Guo Y.-S., Wu B., An Q.-F. (2019). Development of antifouling nanofiltration membrane with zwitterionic functionalized monomer for efficient dye/salt selective separation. J. Membr. Sci..

[cit30] Tan Z., Chen S., Peng X., Zhang L., Gao C. (2018). Polyamide membranes with nanoscale Turing structures for water purification. Science.

[cit31] Jia L., Zhang X., Zhu J., Cong S., Wang J., Liu J., Zhang Y. (2019). Polyvinyl alcohol-assisted high-flux thin film nanocomposite membranes incorporated with halloysite nanotubes for nanofiltration. Environ. Sci.: Water Res. Technol..

[cit32] Yao L., He C., Chen S., Zhao W., Xie Y., Sun S., Nie S., Zhao C. (2019). Codeposition of Polydopamine and Zwitterionic Polymer on Membrane Surface with Enhanced Stability and Antibiofouling Property. Langmuir.

[cit33] Liu Y., Lin B., Liu W., Li J., Gao C., Pan Q. (2018). Preparation and characterization of a novel nanofiltration membrane with chlorine-tolerant property and good separation performance. RSC Adv..

[cit34] Li P., Wang Z., Yang L., Zhao S., Song P., Khan B. (2018). A novel loose-NF membrane based on the phosphorylation and cross-linking of polyethyleneimine layer on porous PAN UF membranes. J. Membr. Sci..

[cit35] Ji Y.-L., Qian W.-J., An Q.-F., Huang S.-H., Lee K.-R., Gao C.-J. (2019). Mussel-inspired zwitterionic dopamine nanoparticles as building blocks for constructing salt selective nanocomposite membranes. J. Membr. Sci..

[cit36] Shen K., Cheng C., Zhang T., Wang X. (2019). High performance polyamide composite nanofiltration membranes via reverse interfacial polymerization with the synergistic interaction of gelatin interlayer and trimesoyl chloride. J. Membr. Sci..

[cit37] Ang M. B. M. Y., Ji Y.-L., Huang S.-H., Tsai H.-A., Hung W.-S., Hu C.-C., Lee K.-R., Lai J.-Y. (2017). Incorporation of carboxylic monoamines into thin-film composite polyamide membranes to enhance nanofiltration performance. J. Membr. Sci..

[cit38] Li Q., Liao Z., Fang X., Wang D., Xie J., Sun X., Wang L., Li J. (2019). Tannic acid-polyethyleneimine crosslinked loose nanofiltration membrane for dye/salt mixture separation. J. Membr. Sci..

[cit39] Zhang N., Jiang B., Zhang L., Huang Z., Sun Y., Zong Y., Zhang H. (2019). Low-pressure electroneutral loose nanofiltration membranes with polyphenol-inspired coatings for effective dye/divalent salt separation. Chem. Eng. J..

[cit40] Zhang J., Yang L., Wang Z., Yang S., Li P., Song P., Ban M. (2019). A highly permeable loose nanofiltration membrane prepared via layer assembled in situ mineralization. J. Membr. Sci..

[cit41] Guo L., Yang Y., Wang Y. (2019). Single-step coating of polyethylenimine on gradient nanoporous phenolics for tight membranes with ultrahigh permeance. J. Membr. Sci..

[cit42] Ma M.-Q., Zhang C., Zhu C.-Y., Huang S., Yang J., Xu Z.-K. (2019). Nanocomposite membranes embedded with functionalized MoS_2_ nanosheets for enhanced interfacial compatibility and nanofiltration performance. J. Membr. Sci..

[cit43] Zhai Z., Jiang C., Zhao N., Dong W., Lan H., Wang M., Niu Q. J. (2018). Fabrication of advanced nanofiltration membranes with nanostrand hybrid morphology mediated by ultrafast Noria–polyethyleneimine codeposition. J. Mater. Chem. A.

[cit44] Jiang C., Tian L., Zhai Z., Shen Y., Dong W., He M., Hou Y., Niu Q. J. (2019). Thin-film composite membranes with aqueous template-induced surface nanostructures for enhanced nanofiltration. J. Membr. Sci..

[cit45] Freger V. (2005). Kinetics of film formation by interfacial polycondensation. Langmuir.

[cit46] Jin J., Liu D., Zhang D., Yin Y., Zhao X., Zhang Y. (2014). Taurine as an additive for improving
the fouling resistance of nanofiltration composite membranes. J. Appl. Polym. Sci..

[cit47] Zhao Y., Zhang Z., Dai L., Zhang S. (2018). Preparation of a highly permeable nanofiltration membrane using a novel acyl chloride monomer with -PO(Cl)_2_ group. Desalination.

[cit48] Fang L.-F., Zhou M.-Y., Cheng L., Zhu B.-K., Matsuyama H., Zhao S. (2019). Positively charged nanofiltration membrane based on cross-linked polyvinylchloride copolymer. J. Membr. Sci..

[cit49] Feng C., Xu J., Li M., Tang Y., Gao C. (2014). Studies on a novel nanofiltration membrane prepared by cross-linking of polyethyleneimine on polyacrylonitrile substrate. J. Membr. Sci..

[cit50] Wei X., Hong J., Zhu S., Chen J., Lv B. (2017). Structure-performance study of polyamide composite nanofiltration membranes prepared with polyethyleneimine. J. Mater. Sci..

[cit51] Mänttäri M., Pihlajamäki A., Nyström M. (2006). Effect of pH on hydrophilicity and charge and their effect on the filtration efficiency of NF membranes at different pH. J. Membr. Sci..

[cit52] Xu P., Wang W., Qian X., Wang H., Guo C., Li N., Xu Z., Teng K., Wang Z. (2019). Positive charged PEI-TMC composite nanofiltration membrane for separation of Li^+^ and Mg^2+^ from brine with high Mg^2+^/Li^+^ ratio. Desalination.

[cit53] Zhang X., Lv Y., Yang H. C., Du Y., Xu Z. K. (2016). Polyphenol Coating as an Interlayer for Thin-Film Composite Membranes with Enhanced Nanofiltration Performance. ACS Appl. Mater. Interfaces.

[cit54] Chen M., Xiao C., Wang C., Liu H., Huang N. (2018). Preparation and characterization of a novel thermally stable thin film composite nanofiltration membrane with poly(m-phenyleneisophthalamide) (PMIA) substrate. J. Membr. Sci..

[cit55] Zhao J., Su Y., He X., Zhao X., Li Y., Zhang R., Jiang Z. (2014). Dopamine composite nanofiltration membranes prepared by self-polymerization and interfacial polymerization. J. Membr. Sci..

[cit56] Yuan S., Li J., Zhu J., Volodine A., Li J., Zhang G., Van Puyvelde P., Van der Bruggen B. (2018). Hydrophilic nanofiltration membranes with reduced humic acid fouling fabricated from copolymers designed by introducing carboxyl groups in the pendant benzene ring. J. Membr. Sci..

[cit57] Xu Z., Liao J., Tang H., Li N. (2018). Antifouling polysulfone ultrafiltration membranes with pendent sulfonamide groups. J. Membr. Sci..

[cit58] Wu M., Ma T., Su Y., Wu H., You X., Jiang Z., Kasher R. (2017). Fabrication of composite nanofiltration membrane by incorporating attapulgite nanorods during interfacial polymerization for high water flux and antifouling property. J. Membr. Sci..

