# Supplementary material for: Preparation of a novel zwitterionic striped surface thin-film composite nanofiltration membrane with excellent salt separation performance and antifouling property
Source: RSC Adv. 2020 Apr 23;10(27):16168–78. doi: 10.1039/d0ra00480d (PMC9052886; doi:10.1039/d0ra00480d)
Supplement: RA-010-D0RA00480D-s001 [file RA-010-D0RA00480D-s001.pdf]

Electronic Supplementary Material (ESI) for RSC Advances.
This journal is © The Royal Society of Chemistry 2020

Supplementary Materials

for

Preparation of a novel zwitterionic striped surface thin-film composite nanofiltration membrane with excellent salt separation performance and antifouling property

Bo Lin[a,b] Huifen Tan[b] Wenchao Liu[b] Congjie Gao[a,c*] Qiaoming Pan[b]
a. Second Institute of Oceanography of the State Oceanic Administration, Hangzhou 310012, China;
b. China National Bluestar (Group) Co., Ltd, Hangzhou 310012, China;
c. Zhejiang University of Technology, Hangzhou 310014, China.

This supporting information contains the following:

S1. The detailed fabrication process was illustrated in support information

S2. The Fig. of the effect of preparation condition in membrane condition

S1. The detailed fabrication process was illustrated in support information

In detail, the PSF UF membrane was fixed with an epoxy resin frame, immersed in DI water. The TA solution was prepared by dissolving TA in phosphate buffer solution (pH = 8.0), followed by mixing with a taurine aqueous solution and PEI aqueous solution. IP was subsequently performed by soaking the membranes in an organic solution of 0.1% weight per volume (w/v) TMC in isopar L. After 1 min reaction time, the excess solution was removed, and the membranes were further dried at 70 ^{o}C in a

blast oven. Finally, the obtained TFC NF membrane was washed with DI water and soaked in DI water for use.

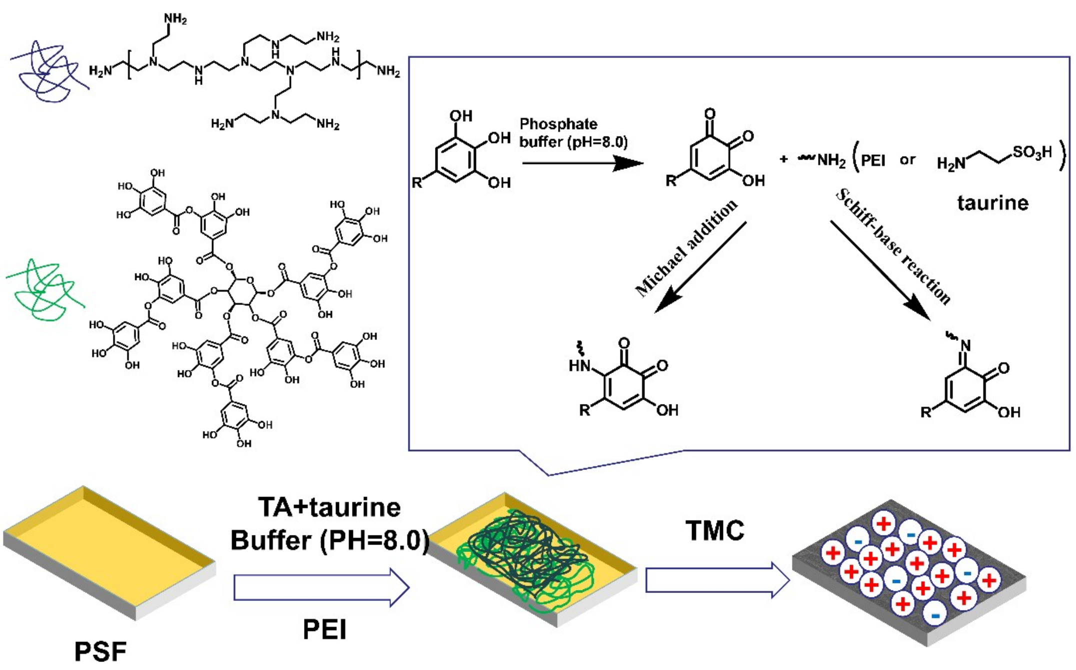

Fig. S1 The process of membrane preparation and the proposed mechanism of the reaction between TA, PEI, and taurine.

S2. The Fig. of the effect of preparation condition in membrane condition

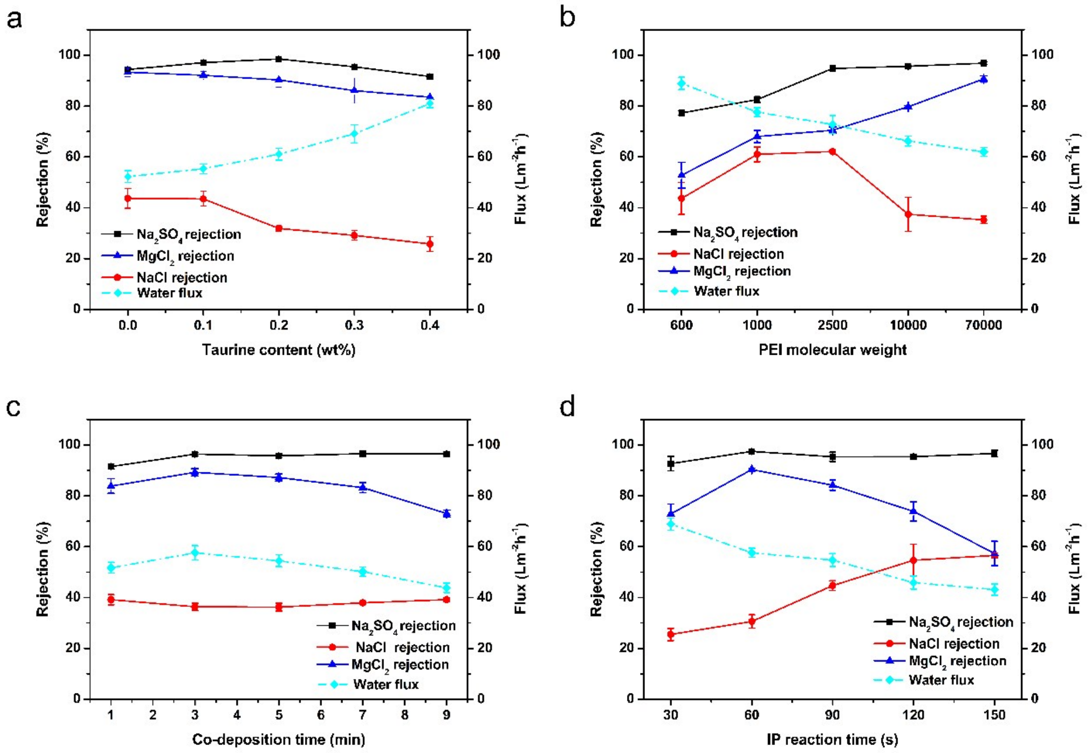

Fig. S2 The effect of preparation condition in membrane condition: (a) the effect of the taurine content on the membrane performance (b) the effect of the co-deposition time on membrane performance (c) the effect of the molecular weight of PEI on membrane performance(d)the effect of the IP reaction time on membrane performance.
